# Factors, Mechanisms, and Kinetics of Spontaneous Emulsification for Heavy Oil-in-Water Emulsions

**DOI:** 10.3390/molecules29132998

**Published:** 2024-06-24

**Authors:** Jinhe Liu, Yao Li, Zengmin Lun, Yuhui Zhang, Pujiang Yang, Xinyu Tang, Qingxuan Zhang

**Affiliations:** 1 College of Chemistry and Chemical Engineering, China University of Petroleum, Qingdao 266580, China; liyao172022@163.com (Y.L.); 19960026@upc.edu.cn (Y.Z.); 19900020@upc.edu.cn (P.Y.); s23030031@s.upc.edu.cn (X.T.); zhangqx@upc.edu.cn (Q.Z.); 2 Experimental Research Center, Sinopec Petroleum Exploration and Production Research Institute, Beijing 100083, China; lunzm.syky@sinopec.com

**Keywords:** heavy oil, spontaneous emulsification, emulsion, in situ surfactant, mechanism, kinetics

## Abstract

In challenging reservoirs where thermal recovery falls short, cold or chemical oil recovery methods are crucial. Spontaneous emulsification (SE), triggered by gentle disturbance, significantly enhances oil recovery. In elucidating SE mechanisms and kinetics, SE processes via direct contact between oil and aqueous phases without stirring were conducted. The effects of temperature, emulsifier concentration, pH, NaCl concentration, and the oil-to-water ratio on SE were investigated through droplet size analysis and turbidity measurements. Furthermore, the emulsification mechanism and derived emulsification kinetics based on turbidity data were obtained. The results underscore the feasibility of SE for oil–water systems, reducing viscous and capillary resistances without agitation. The emulsified oil mass increased with the temperature, pH, and aqueous-to-oil phase volume ratio while decreasing with the NaCl concentration. In this study, for GD-2 crude oil, the optimal emulsified oil amount occurred at a betaine surfactant (BetS-2) emulsifier concentration of 0.45%. Microscopic photo analysis indicated narrow particle size distributions and small droplets, which remained stable over time under various experimental conditions. A combined SE mechanism involving ultralow interfacial tension, interfacial turbulence due to Marangoni effects, and “diffusion and stranding” due to in situ emulsifier hydrophilicity, was speculated. Additionally, an analogous second-order kinetic equation for SE was proposed, indicating exceptional correlation with calculated and experimentally measured values. This study offers theoretical insight for enhancing oil recovery in chemical and cold production of heavy oil in oilfields.

## 1. Introduction

Enhanced oil recovery (EOR) through the in situ formation of oil-in-water emulsions in heavy oil cold production technology has garnered increasing interest from the petroleum industry [1,2,3,4,5,6,7]. Emulsions, comprising mixtures of immiscible liquids with droplets of one phase dispersed within the other, are crucial in oil recovery processes. The prevalent emulsions in oil recovery are typical of the water-in-oil type [8], naturally occurring through crude oil underground migration facilitated by in situ generated or injected emulsifiers within the reservoir [2,9]. However, the high viscosity of water-in-oil emulsions impedes fluid migration in porous media, whereas low viscosity oil-in-water emulsions offer economic viability for oil recovery by enhancing fluid flow through porous media with minimal energy consumption.

The conventional preparation of oil-in-water and water-in-oil emulsions often relies on extra energy-intensive methods, such as high-shear mixers, high-pressure homogenizers, and sonicators [5,10,11], generally called high-energy emulsification. However, these methods are unsuitable for underground implementation in oil production. Thus, spontaneous emulsification (SE) methods, which require no external energy supply or involve low-energy input, such as phase inversion temperature (PIT) [5,12,13,14], phase inversion composition (PIC) [5,12,15,16], solvent diffusion [5,12,17], and Ouzo [5,18], have garnered significant attention from EOR researchers [19,20,21].

Studies have shown that the structure and concentration of surfactants, the composition of the oil-water phase, the addition of non-aqueous solvent and co-surfactant, salinity and pH value, temperature, liquid–liquid bulk phases and their interface, and other factors will affect the spontaneity of the emulsification process [22,23,24,25]. A study by Ying Yang [26] demonstrated that ultrafine oil-in-water emulsions can be produced through high-energy or SE methods, with the latter requiring simple mixing, albeit at higher surfactant-to-oil ratios to achieve droplets with sizes <100 nm. J. Komaiko [27] and Ostertag [28] studied the effects of different surfactant-to-oil ratio (SOR), different surfactants, oil varieties, and surfactant locations on droplet particle size in SE.

The results showed that at higher SORs, the droplet obtained with the non-ionic surfactant Tween 80 was minimal, and the droplet formed when the surfactant was initially dispersed in the oil phase rather than the aqueous phase. Jiang [29] investigated SE induced by a surfactant polymer mixture system composed of hydrophobically modified polyvinyl alcohol (HPVA) and cetyltrimethylammonium bromide (CTAB). The effect of SE was found to be optimal at a concentration of 3000 mg/L and a mass ratio of 7:3 (HPVA: CTAB). Compared to injected surfactants, in situ surfactants can aggregate quickly and precisely at the oil–water interface to achieve more efficient emulsification [30]. Some dual surfactants, i.e., mixtures of two surfactant types, exhibit enhanced effectiveness due to synergistic effects [31,32]. Andersen et al. [33] demonstrated through infrared spectroscopic analysis that carboxylic acids concentrated at the crude oil/water interface. Rostami et al. [34] claimed that microdispersion formation depends on factors, such as experimental conditions and oil characteristics.

Theoretically, emulsification is a thermodynamic non-spontaneous process. However, even slight disturbance energy provided by interfacial turbulence can induce oil-in-water emulsification, known as SE [35]. The mechanism of SE remains incompletely understood [36], with different systems potentially emulsifying through diverse mechanisms or combinations thereof depending on system properties. The main mechanisms involved in this study are: interfacial turbulence, diffusion and stranding, negative interfacial tension, phase inversion, and formation and swelling of water/surfactant aggregates [35]. Studies [37,38] have shown that obvious self-originating emulsions may be formed near oil–aqueous phase interfaces. Chemical potential energy gradients, in liquid–liquid systems without equilibrium, are substantial for facilitating interfacial SE without the aid of external energy inputs and surfactants [39]. Research by Duboué et al. [40] and Beyranvand et al. [41] speculated that water molecules diffuse from the water reservoir into the oil phase, creating droplets simultaneously fed by hydrosoluble “osmogeneous” species, thereby inducing an osmotic pumping of water molecules into microdroplets. Davis et al. [42] observed that the mechanism of SE depended on the surfactant type; nano-particle emulsion (NPE) emulsified via micelle swelling, and SDBS emulsified via nucleation and growth. Zheng et al. [43] suggested that SE may be influenced by the combination of ultralow interfacial tension, Span-80 micelle expansion, and interfacial turbulence caused by the Marangoni effect.

Spontaneous emulsions are characterized in terms of their morphology and the size distribution of droplets [30,35,43], and their emulsifying ability is evaluated through their dynamic evolution [1,30,35,40,43]. Given the evolution data with time, the kinetics of the spontaneous emulsion formation have been measured. Turbidity, typically measured, has been utilized for evaluating emulsion stability [44,45]. Linke [46] systematically studied the factors affecting turbidity in beverage emulsions and observed that particle size is the most significant factor. Shinoda [47] investigated the creaming of oil-in-water (O/W) emulsion by measuring turbidity through ultraviolet–visible (UV-vis) spectrophotometry and found that turbidity decreased with time. The coalescence and/or solubilization kinetics of oil in microemulsion droplets were evaluated by analyzing the temporal distribution of turbidity [48].

Most emulsification kinetics focus on the intermediate steps of the high-energy emulsification process, such as droplet rupture, droplet coalescence, surfactant diffusion, and more [49,50,51]. However, relatively little work on kinetic modeling of emulsification, particularly SE, has been carried out. Toor [52] investigated the influence of a lipophilic surfactant and two water-soluble surfactants on the SE kinetics of aqueous pendant drops in paraffin oil. It was observed that three specific kinetic regimes were mediated by the Span-80 concentration, where SE kinetics turned faster (or slower) upon adding CTAB or sodium dodecyl sulfate (SDS). Karimi [41] studied the spontaneous formation of emulsions in low salinity water (LSW) and observed that emulsions formed within a week, reaching a maximum approximately after 10 days. The rate was controlled under diffusion-induced osmosis imbalance conditions in LSW injection. Zabar [53] investigated the rate of heavy hydrocarbon SE in a non-ionic surfactant solution and proposed a kinetic model based on a diffusion-controlled mechanism, correlating with experimental data. Santana–Solano [54] calculated the rate of interfacial area production utilizing droplet growth at the SE water/oil lipophilic surfactant interface. The results showed that their growth conforms to a power law for a certain range of surfactant concentrations. Sitnikova [55] studied the SE and the growth of droplet in trans-anethol/water/ethanol solutions and observed that droplet growth was controlled through Ostwald ripening, with kinetics proving a ripening saturation limit at a droplet radius of ~1.5 µm.

To further elucidate the SE mechanism and kinetics, the effects of several factors on the SE of crude oil in water were investigated. Furthermore, emulsification kinetics based on turbidity data were obtained.

## 2. Results and Discussions

### 2.1. Interfacial Tensions (IFTs) of the SE System

**Figure 1:**
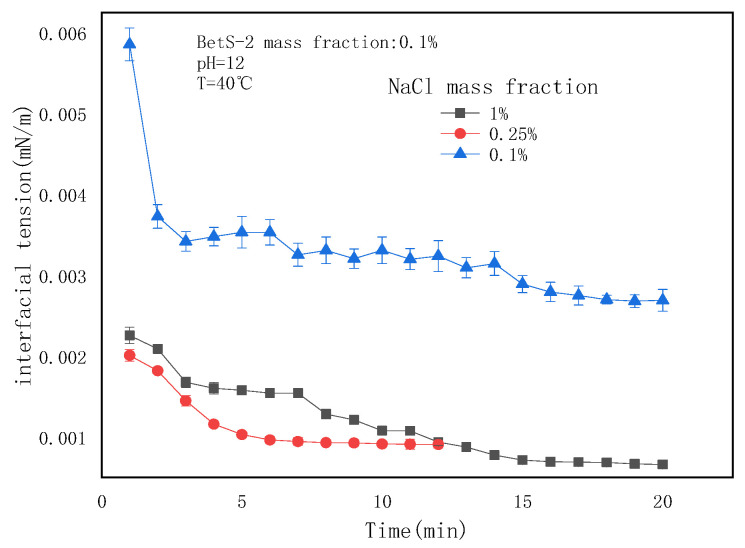
The effects of the NaCl mass fraction on IFT.

**Figure 2:**
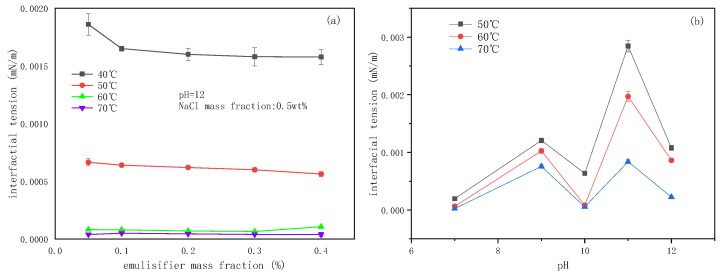
(**a**) The effects of the BetS-2 mass fraction and (**b**) pH on interfacial tension.

The emulsification resistance of the SE system primarily includes the cohesive force of the dispersed phase and the deformation resistance of droplets due to the IFT. The mixture of heavy oil and toluene (3:1, *v*/*v*) reduced the viscous resistance of the oil phase. Meanwhile, the reduction of deformation resistance was achieved upon adjusting the IFT to an extremely low value. The IFTs under different conditions were obtained and are illustrated in Figure 1 and Figure 2.

The dynamic IFT at different salt concentrations (Figure 1) exhibited a slow decrease with time, indicating sluggish diffusion of the emulsifier to the interface or the formation of a viscous film. The IFT decline rate with time remained substantially unaffected by the salt concentrations. However, the equilibrium IFT decreased and then stabilized with NaCl concentrations exceeding 0.1wt%, indicating that salt addition facilitated IFT reduction.

The IFT decreased slightly with increasing emulsifier concentrations, particularly at higher temperatures (Figure 2a). Under the experimental conditions, the achievement of ultra-low IFT facilitated the SE process. The complex relationship between pH and IFT originated from the amphoteric nature of betaine emulsifiers (Figure 2b).

### 2.2. The Effect of Temperatures on the SE Process

The main indexes to evaluate SE efficiency are the quality of emulsified crude oil and the oil droplet particle sizes of O/W emulsions. The influences of temperature, salt concentration, pH, and emulsifier dosage on the SE capacity were investigated.

**Figure 3:**
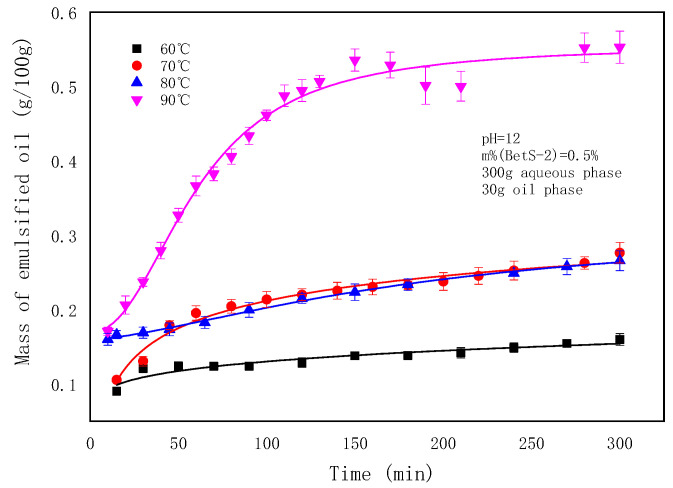
The effect of temperature on emulsified oil mass.

**Figure 4:**
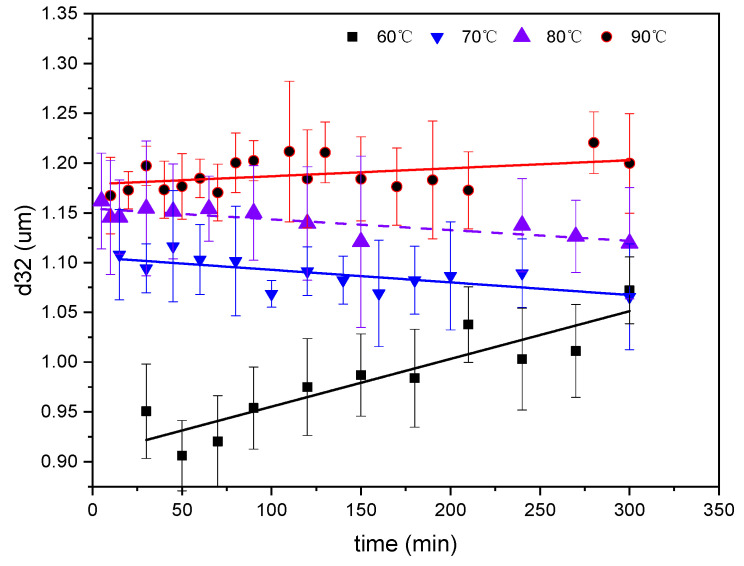
Evolution of emulsion particle size over time at different temperatures.

The emulsification process was carried out under the condition of a 1:10 oil–water mass ratio, in which the concentration of water phase emulsifier was 0.6wt%, pH = 12, and the oil phase ratio (m (GD-2 oil)/m (toluene)) was 1:3, at different temperatures. The turbidity of the emulsion at different times was measured, and the mass of emulsified oil in the emulsion was obtained according to the standard curve. The effects of temperature on the emulsified oil mass and particle sizes are depicted in Figure 3 and Figure 4, respectively.

**Figure 5:**
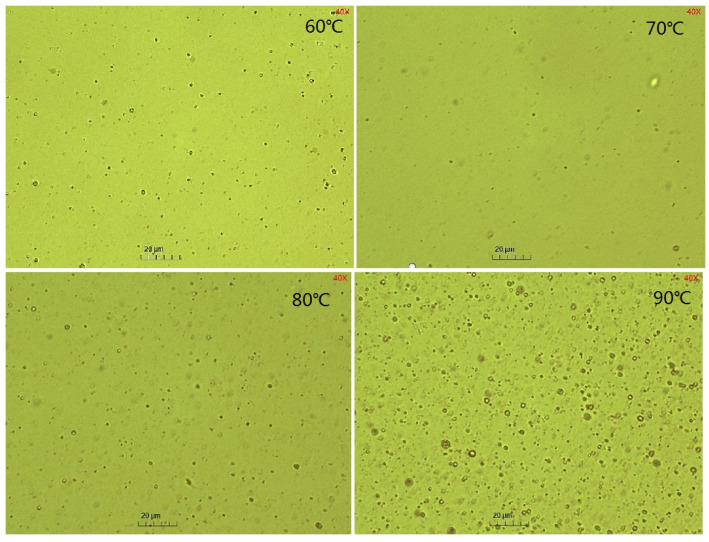
Microscopic images of emulsion at different temperatures.

In Figure 3, it can be observed that the increase in temperature elevated the emulsified oil mass in the emulsion from 0.16–100 g at 60 °C to 0.55–100 g at 90 °C. The emulsified oil mass gradually increased, correlating with the emulsification time at different temperatures. In Figure 4 and Figure 5, it can be observed that the particle size of the emulsion slightly increased with time at 60 °C. However, slight variations occurred with time as the temperature exceeded 60 °C. Larger particle sizes and higher particle numbers correspond to higher emulsification temperatures in Figure 3 and Figure 4. Temperature affected the surface tension by decreasing the solvation of the hydrophilic groups of the emulsifier, rearranging emulsifier molecules more closely on the oil–water interface, reducing the phase viscosity, and enhancing the emulsifier diffusion to the interface [56,57]. Figure 2a shows that ultra-low IFT was obtained at the experimental temperature, ensuring a smooth SE process. However, as the temperature increased, surfactant molecules were more likely to aggregate on the surface, and the concentration gradient of surfactants increased. When the water phase interface is affected by temperature or concentration gradients, Marangoni instability occurs [58]. Surfactant concentration gradients and Marangoni flows were enhanced with increasing temperature.

The results indicate that temperature facilitated SE probably due to the increased diffusion and water solubility of the in situ emulsifier formed within the oil phase or the increased interfacial turbulence. It was observed that the particle sizes increased with the temperature, which contradicts some results in this study. Saberi et al. [59] found that the particle size decreased with increasing holding temperature at several surfactant concentrations due to the decreased emulsion viscosities. Furthermore, increased surfactant water solubility facilitated the formation of fine droplets using the SE method.

### 2.3. The Effect of Emulsifier Concentrations on the Emulsification Process

**Figure 6:**
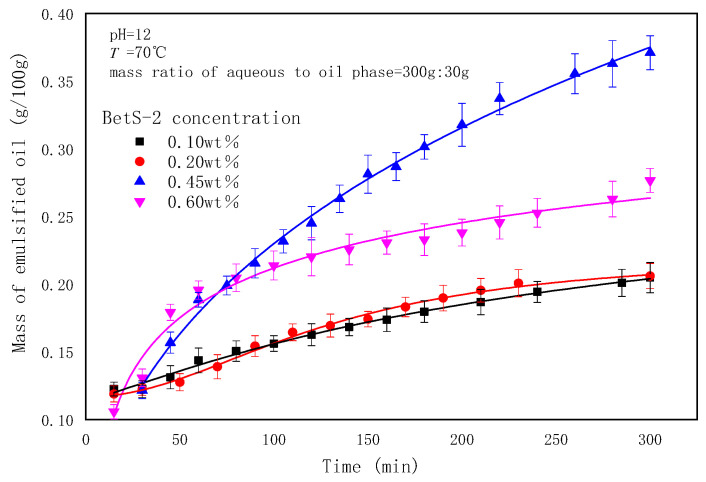
The effect of emulsifier concentrations on emulsified oil mass.

**Figure 7:**
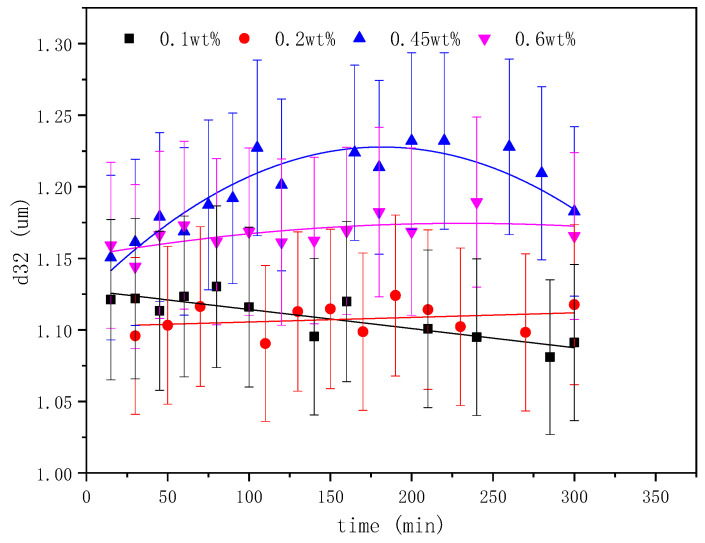
Evolution of emulsion particle size over time at different BetS-2 amounts.

**Figure 8:**
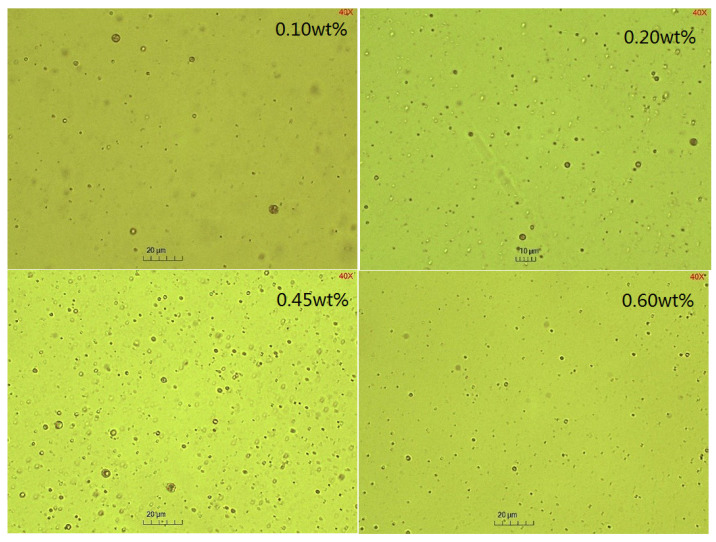
Microscopic images of emulsion with different BetS-2 amounts.

Two types of emulsifiers were investigated in this study. The BetS-2 was artificially added to the water phase and in situ generated emulsifiers to the oil phase, where acidic components in GD-2 crude oil, such as fatty acids, naphthenic acids, and others, were converted into organic acidic salts under alkaline conditions. The effect of the BetS-2 concentrations in the aqueous phase on the SE process was investigated (Figure 6, Figure 7 and Figure 8).

The amount of emulsified oil increased with emulsification time, but it initially increased and then decreased with emulsifier concentrations. The exceptional emulsification efficiency was obtained at 0.45wt% emulsifier concentrations. It was observed that a higher emulsifier concentration was not suited to the SE process, correlating with the high energy emulsification and field situations [1]. At emulsifier concentrations other than 0.45%, the particle size (basically between 1.08 um and 1.25 um) did not vary significantly with emulsification time (considered essentially unchanged within the error range). However, the particle size increased initially and then decreased as the emulsifier concentration was 0.45%, at which point a lesser degree of droplet size uniformity occurred in the SE process.

Studies have shown that the surfactant-to-water ratio influences the formation and droplet size of emulsions prepared using SE methods. The results indicated that there was an optimum emulsifier concentration in the SE process, and the particle size increased with surfactant concentration addition. Mehrnia et al. [60] also found the optimum surfactant concentration for enhancing microemulsions using non-ionic surfactants, such as Span-80 and polyglycerol polyricinoleate. However, they observed that the particle size decreased initially and then increased with emulsifier concentrations. These different conclusions were attributable to the differences in the emulsification system. The increase in the emulsifier concentration also changed the ratio of in situ emulsifiers to artificially added emulsifiers, thereby affecting the interface competitive adsorption and the emulsion properties.

### 2.4. The Effect of pH on the Emulsification Process

**Figure 9:**
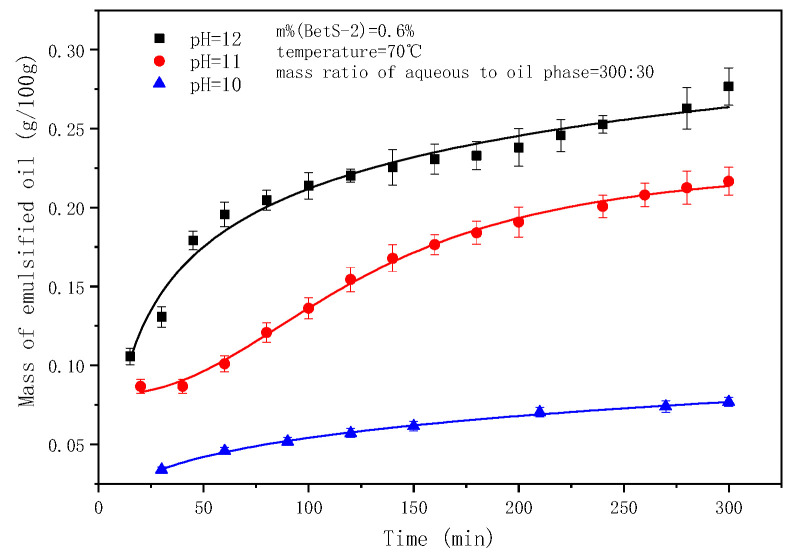
The effect of pH on emulsified oil mass.

**Figure 10:**
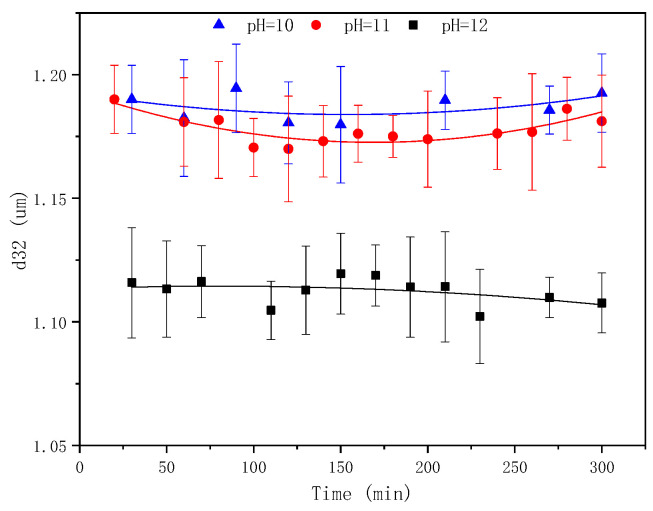
Evolution of emulsion particle size over time at different pH values.

The pre-experiment results indicated that the SE process was slightly triggered when the pH of the water phase was ≤9. Thus, in this study, experiments were conducted with pH = 10–12. The emulsified oil mass and droplet sizes at different times are shown in Figure 9 and Figure 10, respectively.

**Figure 11:**
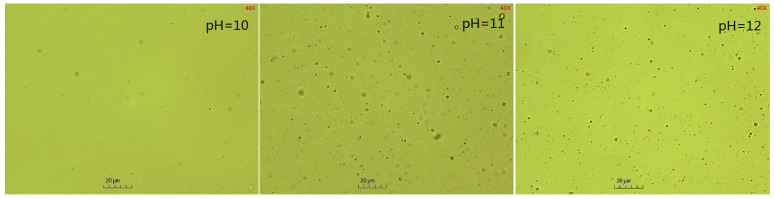
Microscopic images of emulsion at different pH values.

The pH plays a crucial role in the SE process of crude oil emulsification into water to form an oil-in-water emulsion [61]. Notably, the alkalis reacts with the indigenous acids (such as fatty acids and naphthenic acids) in crude oil to form in situ surfactants. The higher the pH value, the greater the content of acid deprotonation into the in situ emulsifier, facilitating the oil component diffusion through the oil–water interface to the water phase, forming oil droplets. The competitive adsorption of the artificial emulsifier BetS-2 and the in situ emulsifier formed a solid interfacial film on the oil droplet surface, ensuring the stability of the droplets. Figure 9 shows that as the pH increased from 10 to 12, the amount of emulsified oil augmented 3.8 times from 0.077 g/100 g to 0.27 g/100 g over a 5-h emulsification period. Thus, the high pH was responsible for the high amount of emulsified oil. It was observed that the amount of emulsified oil slightly changed with time. Additionally, as the emulsification process progressed, the number of in situ emulsifiers generated gradually decreased due to the decreasing pH in the solution, reducing the acidic composition in the oil phase. Thus, the ability of in situ emulsifiers to carry oil components into the aqueous phase was minimized. Figure 10 and Figure 11 show that the pH also affected the particle size of the emulsion. The smaller the pH, the larger the emulsion particle size, indicating that a high pH was conducive to smaller droplets and more stable kinetically emulsions. Additionally, it was observed that emulsification primarily occurred due to the diffusion of the in situ emulsifier from the oil to aqueous phase, owing to the formation of interfacial turbulences [62] or diffusion and the stranding mechanism. BetS-2 emulsifier exhibited no effect on the formation of droplets, and it was mainly used to stabilize the droplets. This indicates that BesT-2 was an excellent emulsifier in high-energy emulsification. Li et al. [63] reached the same conclusion, observing that crude oil was spontaneously dispersed into the water phase under static conditions with an alkali addition exceeding 0.1%. Riehm et al. [64] also revealed a phenomenon where the dispersion medium containing a high concentration of Tween 80 can promote the formation of oil-in-water SE. This suggests that the “diffusion and fixation” mechanism may be the most reasonable theory to explain the formation of oil-in-water SE. During this process, the diffusion behavior of Tween 80 at the oil–water interface plays a crucial role, as it can strongly promote the emulsification process of oil droplets. Pei et al. [65] reached opposite conclusions, obtaining W/O emulsion by an in situ emulsifier during the alkaline-flooding process. These differences probably stemmed from the excessive cohesion of heavy oil utilization.

### 2.5. The Effect of NaCl Concentrations on the Emulsification Process

**Figure 12:**
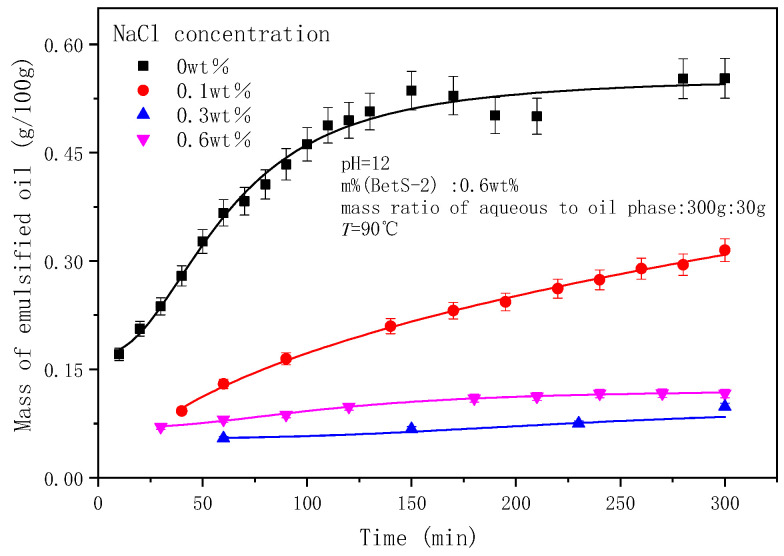
The effect of NaCl concentrations on emulsified oil mass.

**Figure 13:**
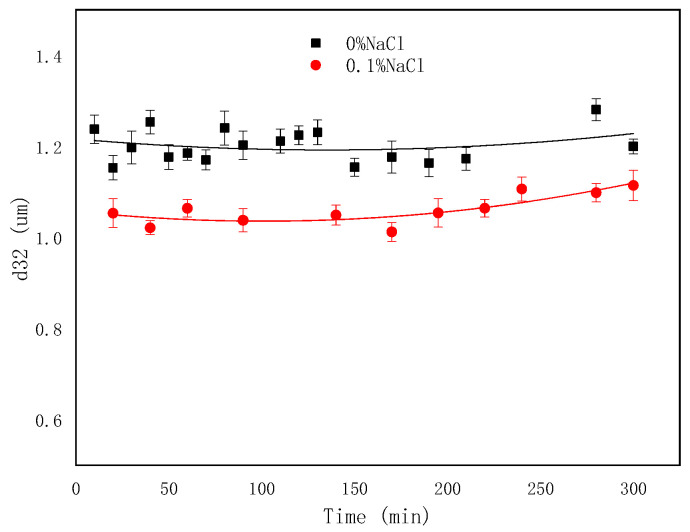
Evolution of emulsion particle size over time at different NaCl contents.

**Figure 14:**
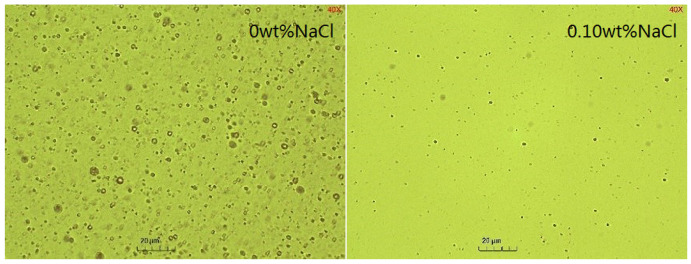
Microscopic images of emulsion at different NaCl contents.

It is shown in Figure 12 that as the NaCl concentration increased, the amount of emulsified oil decreased, indicating that salt inhibited the SE process. The sizes of droplets decreased with NaCl addition and remained almost unchanged over time (Figure 13 and Figure 14). The effect of salts on the droplet size is correlated with the findings of Zabar [66]. The complex effects of salinity on the SE process have been observed by researchers. As the salt concentration in the oil-in-water (O/W) emulsion increases, the self-aggregation tendency of the emulsion strengthens, and in completely salt-free samples, only a slight stabilizing effect is shown [43]. Silva et al. [67] demonstrated that adding salt to the aqueous phase of the system can effectively reduce the formation of droplets. Su et al. [68] revealed the promoting and inhibiting effects on the formation of nanoemulsion of the NaCl concentration in the ranges of 0.2–0.4 mM and 0.6–0.8 mM, respectively. Ultra-low interfacial tension was achieved with NaCl addition (Figure 1), however, the difficulty of emulsification with the addition of NaCl proved that IFT was not the root cause of the SE [67]. It probably originated from the increased hydrophobicity of the surfactant molecules through the dehydration of the emulsifier hydrophilic head with an increasing salt concentration [69]. This made it difficult for the in situ emulsifier molecules to migrate to the aqueous phase, forming oil droplets.

### 2.6. The Effect of the Mass Aqueous-to-Oil Phase Ratio on Emulsification

**Figure 15:**
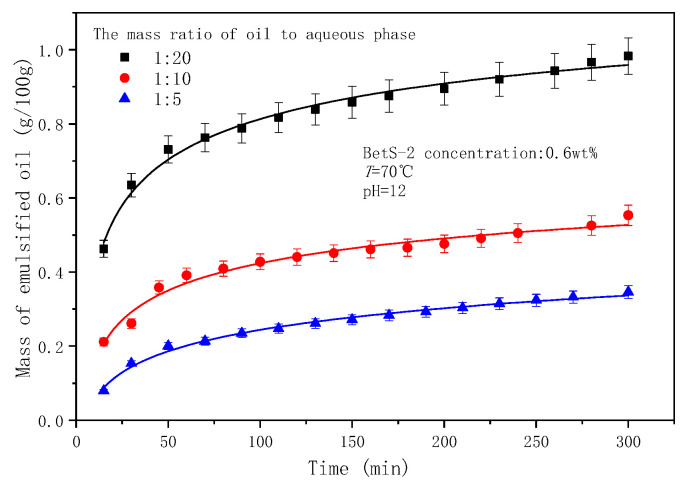
The effect of the ratio of aqueous to oil phase on mass of emulsified oil.

**Figure 16:**
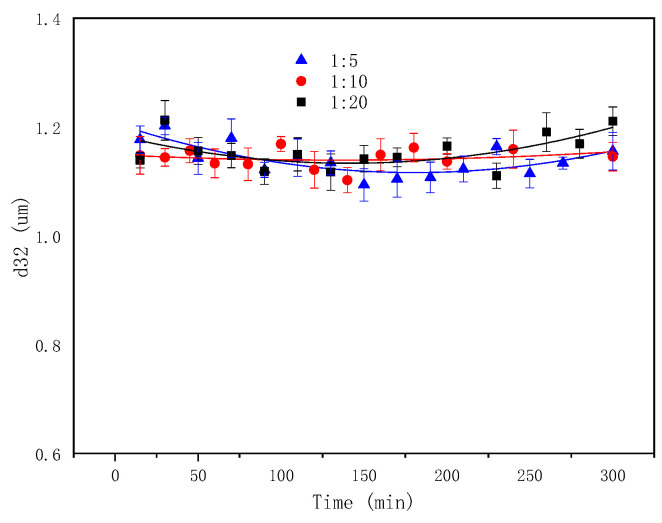
Evolution of emulsion particle size with time at different RAOs.

**Figure 17:**
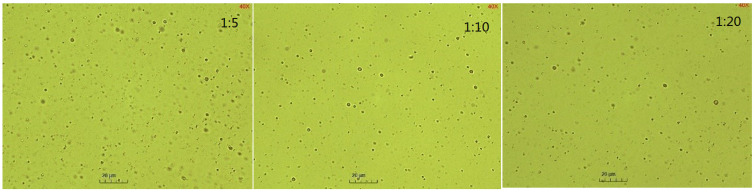
Microscopic images of emulsion at different RAOs.

Higher contents of oil were emulsified into water (Figure 15), but the droplet sizes slightly changed with an increasing aqueous-to oil-phase ratio (RAO) (Figure 16 and Figure 17). This indicates that the RAO exhibited a significant impact on the SE process, with a negligible effect on the properties of the droplet population. The contact surface between the oil phase and the water phase of the raw material and the diffusion rate of the in situ emulsifier remained constant when the RAO increased. Therefore, the augmented amounts of emulsified oil originated from the enhanced numbers of in situ emulsifiers with an increasing water phase quantity. It was observed that with increasing amounts of aqueous phase, more organic acids reacted with the base to form more in situ emulsifiers, which carried more oil into the aqueous phase. The effect of the oil–water ratio in this study correlates with that of the number of surfactants in the oil phase (i.e., SOR) in Wang’s work [70]. Wang [70] obtained similar results, which were attributable to the increasing efficiency of the surfactant at the interface as the SOR increased.

### 2.7. Mechanism of Spontaneous Emulsification

Different systems undergo SE through various mechanisms or a combination of mechanisms depending on the property of the system [43]. In this study, the formation of oil droplets in water required overcoming two kinds of resistances: the viscous force of the dispersed phase and the capillary resistance of the interfacial phase, which was achieved by diluting the oil phase and reducing the IFT to an ultra-low level. It was identified that the SE process was not conducted when the pH was <9, indicating that the SE process was triggered at a high pH, forming a substantial amount of in situ emulsifiers. This was attributable to the low content of organic acids in the crude oil. Additionally, emulsifier zones of uneven concentration were located at the interface, leading to interfacial turbulence due to Marangoni effects. Meanwhile, another plausible mechanism for this system was attributable to “diffusion and stranding”, in which the lipophilic acidic components in the oil phase produced a hydrophilic in situ emulsifier through acid–base reactions. The hydrophilic emulsifier carried the oil components across the interface into the aqueous phase, in which the oil droplets were stabilized by the synergistic effect of in situ and factitious Bet-2 emulsifiers, correlating with other studies [64]. Moreover, the rapid change in the solubility of the in situ emulsifier from lipophilic to hydrophilic properties was attributable to the interface turbulence. The particle sizes slightly changed over time during emulsification, indicating the stability of the generated emulsion. It was concluded that a combined effect of the ultralow IFT and interfacial turbulence due to Marangoni effects and “diffusion and stranding” led to the hydrophilicity of the in situ emulsifiers. Thus, the main mechanism of SE was observed in this study. The strong hydrophilic BesT-2 emulsifier alone could not trigger the SE process, mainly due to the BesT-2 molecules migrating across the interface into the oil phase. This indicates that the diffusion of lipophilic emulsifiers from the oil phase to the aqueous phase was suitable for O/W emulsion formation. At high salt concentrations, there was still the Marangoni effect due to the inhomogeneous emulsifier concentration at the interface. The SE process was slow and slightly triggered, indicating that the “diffusion and stranding” mechanism played a crucial role in the combined mechanisms. An important implication of this hypothesis was that the emulsified oil amounts tended to be constant due to diffusion and stranding, depleting in situ surfactants in the oil and complicating the further dispersion of the oil into the aqueous phase.

### 2.8. The Kinetic Equation for the SE Process

**Figure 18:**
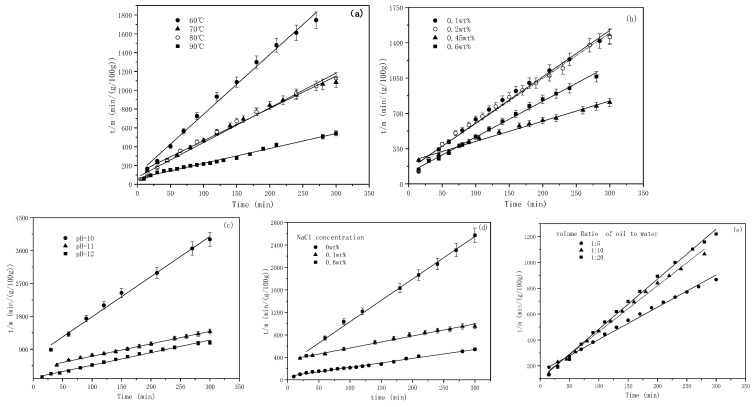
The *t/m* varies as a function of time during the SE process under various emulsification conditions ((**a**): temperature (**b**): concentration (**c**): pH (**d**): NaCl concentration (**e**): oil-water ratio). Solid dots denote the experimental data, while the solid line (dashed line) represents the theoretical curve, as provided by Equation (4).

The emulsification kinetic equation has been obtained typically based on droplet size data [41,54,55] or deduced from a specific elementary step within the complex SE process [53]. In this study, based on the turbidity measurement, the overall kinetic equation for the SE process corresponded to the emulsification rate, which is defined as the rate of change of emulsified oil content over time (Equation (4)). Thus, Equation (4) was employed to fit the SE data at different conditions, ensuring its validity (Figure 18).

In Figure 18, the deviation between the simulated and experimental data is shown. The correlation coefficients under all other experimental conditions surpassed 0.99. This indicates the applicability of the second-order kinetic equation in describing the SE kinetics of GD-2 crude oil diluted with toluene. In this study, compared to kinetic equations governing individual elementary steps of the complex SE dynamic process, the proposed kinetic equation offers superior practicality in oil production.

## 3. Materials and Methods

### 3.1. Materials

This experiment used GD-2 crude oil provided by the Shengli Oilfield. A mixture of oil and toluene (1:3, *v/v*) as the dispersed phase material to lower the viscosity of GD-2 crude oil. The oil viscosity was analyzed using an NTV-T2 temperature controlled viscometer (Shanghai Nirun Intelligent Technology Co., Ltd., Shanghai, China), while the oil density was measured using an AU-300 API petroleum density meter (Hangzhou Jinmai Instrument Co., Ltd., Hangzhou, China). The components of saturates, asphaltenes, resins, and aromatics (SARA) in the oil were measured according to the ASTM D4124-09 standards [71]. In addition, the contents of acidic alkaline components in the asphaltenes was obtained according to the USBM-API standard [72]. The properties and compositions of the oil are detailed in Table 1. The elemental contents of resins and asphaltenes in the crude oil were analyzed using the VARIO EL III elemental analyzer (Elemental Analysensysteme Co., Ltd., Frankfurt, Germany) (Table 2).

Furthermore, BetS-2 was supplied by the Research Institute of Petroleum Exploration and Development (RIPED) of CNPC. Hydrochloric acid was purchased from Sichuan Xilong Chemical Co., Ltd., Chengdu, China. The chemicals, including NaCl, Na_2_CO_3_, NaOH, and toluene, were provided by Sinopharm Chemical Reagent Co., Ltd., Shanghai, China. All compounds were used directly. Purified water (secondary distilled water) was prepared in the laboratory.

### 3.2. Experimental Methods

#### 3.2.1. Spontaneous Emulsification

**Figure 19:**
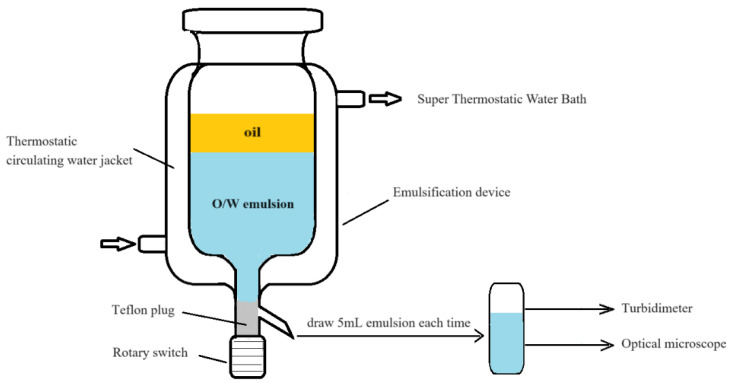
Experimental setup.

The experimental setup for SE is shown in Figure 19.

A standard emulsion was prepared in three steps. First, the aqueous phase was prepared using purified water, BetS-2, and NaCl, the pH value of which was measured using a PHS-3C pH meter (Shanghai Yidian Scientific Instruments Co., Ltd., Shanghai, China) and adjusted utilizing a Na_2_CO_3_ solution due to its buffering capabilities. Second, the oil phase was formed by mixing GD-2 crude oil with toluene in a ratio of 1:2 (mass ratio). Third, the aqueous phase was initially placed into a 400 mL emulsification device. Subsequently, when the aqueous phase was heated to the predetermined temperature, the oil phase was slowly added along the inner wall of the container, ensuring minimal interference with the interface. Droplets of oil-in-water nanoemulsion were formed spontaneously through the oil phase fraction diffusion into the aqueous phase.

#### 3.2.2. Emulsion Turbidity Measurement

Turbidity measurement was employed for the evaluation of emulsion stability as well as the coalescence and/or solubilization kinetics of oil in microemulsion droplets. Various factors, primarily droplet concentrations and droplet sizes, affect emulsion turbidity [46,73]. The study results indicate that the effects of a slight increase in droplet size during emulsification on the turbidity were negligible, and the change in the emulsion turbidity with time was significantly attributable to the emulsion concentration increase. Shinoda [47] also suggested that a higher concentration of an oil component generally results in high turbidity, although several other factors can also affect the turbidity.

In this study, the emulsion turbidity was measured at room temperature using a diffusion turbidimeter (WGZ-2000B) (Shanghai INESA Physico Optical Instrument Co., Ltd., Shanghai, China). The wavelengths of the incident light were 860 nm, at which the light absorption of each component in an emulsion was reasonably neglected because the light detector was at 90° from the incident light. The solution was diluted with the aqueous phase of the emulsion if the emulsion turbidity exceeded the maximum range (2000 NTU) of the turbidimeter, regardless of the emulsion collapse mentioned by Shinoda [47]. The turbidimeter was calibrated using a series of turbidity standards ranging from 0 to 2000 NTU.

#### 3.2.3. Mass of oil Emulsified Measurement

To establish the relationship between the degree of turbidity and the oil-emulsified mass, the emulsification procedure was performed under the conditions of 250 mL of aqueous phase with 1wt% BetS-2, pH = 10 without NaCl, and 50 mL of oil phase at 50 °C. At different times during the emulsification process, ~5 mL emulsion was drawn, and its turbidity was measured. Subsequently, the emulsion was transferred to pre-dried and weighed aluminum foil boxes (*m*_1_) and weighed to obtain the emulsion mass (*m*_2_). The aluminum foil box with the emulsion was placed in a vacuum drying oven (80 °C, 10 kPa) and dried to a constant weight, and the mass (*m*_3_) of the aluminum box and emulsion residue after drying was weighed. The mass concentration *m*/(g/100 g) of the emulsified oil per 100 g of water was calculated using Equation (1).

(1) $$m=\frac{m_{3}-m_{1}}{m_{2}-m_{0}}\times 100\%$$

where *m*_0_ represents the salt and emulsifier content in the dried emulsion.

**Figure 20:**
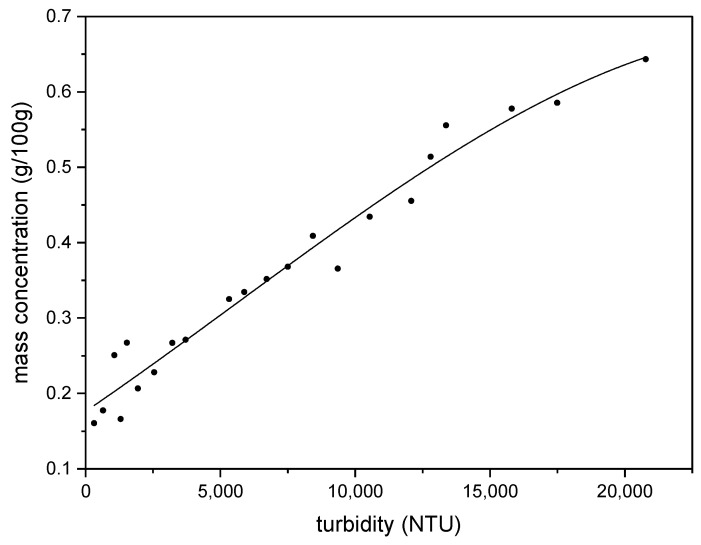
The relationship between turbidity and emulsion concentration.

The standard curve was established with the measured turbidity, and the emulsified oil mass concentration was calculated using Equation (1) (Figure 20). The slight deviation of the standard curve from the linear relationship was due to the particle size and high emulsion concentration [73]. Based on the experimental fact that the particle size did not change much under the different experimental conditions and different times, the influence of particle size on the turbidity can be ignored. Subsequently, the emulsion concentration was obtained from the standard curve according to the emulsion turbidity measurements.

#### 3.2.4. Droplet Size Measurement

The emulsions obtained at different emulsification times were observed via visual examination of the emulsion droplets and recorded using a BA310-T optical microscope with a CCD camera (Motic China Group Co., Ltd., Guiyang, China). The microscopic state of the different O/W emulsions was investigated, and the sizes of the oil droplets were measured using the publicly available software Image J (version 1.8.0). The operators measured each droplet separately to avoid possible errors, which can occur in automated image analysis procedures [1].

Additionally, for each set of five photos of each sample, diameters of at least 2000 droplets were measured to ensure statistical significance in determining the droplet size distribution. The average diameter *d*_32_ was determined based on the measured droplet diameters. In all cases, each experiment was repeated at least three times.

(2) $$d_{32}=\frac{\sum_{i}n_{i}d_{i}^{3}}{\sum_{i}n_{i}d_{i}^{2}}$$

where *x*_i_ is the number of droplets with diameter *d*_i_.

#### 3.2.5. Interfacial Tension (IFT) Measurement

The O/W IFT between the oil phase and chemical aqueous solution was measured using a CNG700 spinning-drop interfacial tensiometer (Beijing Shengwei Technology Co., Ltd., Beijing, China) at a rotating velocity of 5000 rpm. In all cases, a minimum of two measurements were made to obtain the average IFT.

#### 3.2.6. Emulsification Rate and Kinetic Equation

The kinetic equation developed by Liu [1] was adopted in this study and is briefly described as follows: The emulsification rate was defined as *r* = d*m*/d*t*, where *r* is the emulsification rate, g·100 g^−1^ emulsion·s^−1^; and *m* is the mass of emulsified oil (g·100 g^−1^ emulsion) at the emulsification time *t* (min). The process of the emulsification of crude oil into emulsion conforms to the second-order chemical reaction kinetic equation. The rate equation is as follows.

(3) $$\frac{dm}{dt}=k{\left(m_{0}-m\right)}^{2}$$

where *m* is the mass of emulsified oil at time *t*, g·100 g^−1^ water; *m*_0_ is the maximum mass of emulsified oil, g·100 g^−1^ water; *t* is the emulsification time, min; *k* is the emulsification rate constant related to the energy provided through emulsification, emulsification process, crude oil, temperature, pressure, continuous phase, and other factors, g·100 g^−1^·min^−1^.

Equation (3) is integrated with *m* = 0 at *t* = 0 and *m* = *m* at *t* = *t*, then reorganized to obtain Equation (4).

(4) $$\frac{t}{m}=\frac{1}{km_{0}^{2}}+\frac{t}{m_{0}}$$

where *m*_0_ can be obtained from the slope of the linear relation between *t*/*m* and *t,* and slope ^2^/intercept yields the rate constant *k*.

## 4. Conclusions

This study conducted an experimental investigation into the SE of a high-pH surfactant solution upon contact with diluted GD-2 crude oil and toluene. Upon reducing the viscosity through crude oil dilution and decreasing the capillary resistance via ultra-low IFT, the SE process was conducted even without agitation. Emulsification initiation occurred via in situ emulsifiers formed by the acid–base reaction of acidic components in the crude oil at the interface. This study suggests a combined mechanism involving ultralow interfacial tension, interfacial turbulence due to Marangoni effects, and “diffusion and stranding” induced by the hydrophilicity of in situ emulsifiers, with “diffusion and stranding” being the predominant factor. The rate of emulsified oil content and droplet size evolution during SE was characterized by the emulsification efficiency. The emulsified oil mass increased with an increasing temperature, pH, and aqueous-to-oil phase volume ratio, while decreasing with higher NaCl concentrations. Additionally, for GD-2 crude oil, the optimal emulsified oil mass occurred at a BetS-2 emulsifier concentration of 0.45%. The droplet sizes exhibited minimal variation over time under various experimental conditions, indicating single-time droplet formation without subsequent nucleation, growth, dispersion, and reaggregation processes during emulsification. An analogous second-order overall kinetic equation accurately describes the evolution of the emulsified oil mass calculated, with correlation coefficients exceeding 0.99 when compared to the experimental values.

## Figures and Tables

**Table 1:** Contents of SARA of GD-2 crude oil and acidic–basic fractions in asphaltenes.

Density/g·cm^−3^	SARA Fraction Content of GD-2 Oil/%			
	*w* (Saturates)	*w* (Aromatics)	*w* (Resins)	*w* (Asphaltenes)
0.9282	28.79	32.15	28.62	8.97
Viscosity/mPa·s at 50 °C	Acidic–Basic Fraction Content of Asphaltenes/%			
	*w* (Acidic)	*w* (Basic)	*w* (Amphoteric)	*w* (Neutral)
965	7.69	5.68	7.57	76.72

**Table 2:** The elemental contents of SARA fractions of GD-2 oil.

Components	Element Contents/%				
	*w* (C)	*w* (H)	*w* (S)	*w* (O)	*w* (N)
Saturates	85.56	13.59	0.55	0.30	0
Aromatics	85.50	12.82	1.18	0.37	0.13
Resins	85.37	11.49	1.47	0.91	0.76
Asphaltenes	84.15	8.96	3.24	2.17	1.48

## Data Availability

The original contributions presented in the study are included in the article, further inquiries can be directed to the corresponding author.
